# Homogeneous Electrochemical Immunoassay Using a Chemically Modified Electrode

**DOI:** 10.6028/jres.093.138

**Published:** 1988-06-01

**Authors:** Rosanne Kannuck, Jon M. Bellama, Richard A. Durst

**Affiliations:** Department of Chemistry and Biochemistry, University of Maryland, College Park, MD 20742; Center for Analytical Chemistry, National Bureau of Standards, Gaithersburg, MD 20899

An alternative to enzymatic detection and amplification of antigen/antibody interactions is the use of lipid bilayer structures known as liposomes. The concept in using this type of structure is that a specific “marker” is released from within the liposome when an antigen/antibody binding event has taken place on the liposome surface. Amphophilic phospholipid molecules spontaneously aggregate in aqueous media to minimize the interaction free energy per lipid molecule. This aggregation results in the formation of closed bilayer structures consisting of an aqueous internal cavity separated from the external solution by a lipid membrane. If the aqueous solution used in the liposome preparation contains high concentrations of any of a variety of dyes, proteins, or ionic species, the result is that the marker is entrapped within a closed bilayer. The physico-chemical properties of the liposome are governed by the type of marker used, chemical composition of the liposome (the types of amphophiles used), molecular size and charge of the marker species, and membrane surface ligands.

The membrane surface ligands are either antigens or antibodies that have been covalently bound to the surface of certain phospholipid head group entities, such as an amine molecule, and impart the immunogenic properties to the liposome. After a binding event occurs, the liposomes will act as a receptor for complement attack, that is, a series of naturally occurring serum proteins which recognizes the event and lyses (disrupts) the membrane.

Recent work in this laboratory has demonstrated the feasibility of encapsulating high concentrations (50 mmol/L) of potassium ferrocyanide in liposomes and detecting the released marker by differential pulse voltammetry [1]. We have found, however, that modification of the electrode is necessary to overcome the interferences encountered in the immune lysis of liposomes and to enhance the oxidative peak current of the liposome-released ferrocyanide. This latter step is accomplished by electrostatically binding the released marker at the electrode surface via a technique described by Whiteley and Martin [2] as ion-exchange voltammetry (IEV). The ferrocyanide ion is electrostatically bound within a siloxane polymer film on the electrode surface by the exchange with iodide present in the film as a methylpyridinium iodide (fig. 1).

A film thickness of 1.2 *μ*m was shown to be adequate coverage for electrode protection. Figure 2 illustrates the ion-exchange capabilities of these electrodes in serum matrices, where each data point is the average of three different electrodes in 1 × 10^−5^ mol/L ferrocyanide (final concentration). Peak currents measured at a bright Pt (0.635 cm^2^) electrode for this concentration are typically on the order of 0.1 *μ*amp. Therefore, it is seen that even with 60% serum in the matrix, the signal obtained is five times greater at the modified electrode.

Our research utilizes liposomes immunologically sensitized by the addition of 1 mole percent dinitrophenol-capped phosphatidylethanolamine (DNP-cap PE) to liposomes prepared from a mixture of dimyristoylphosphatidylcholine (DMPC), cholesterol, and dicetyl phosphate (DCP) in a 5:4:1 mole ratio. Using this technique, we have been able to demonstrate an analysis for ani-DNP IgM using the DNP-fixed liposomes and measuring the ferrocyanide signal as a secondary response for the presence of IgM.

The amount of complement greatly affects the permeability of the lipid bilayer. We have found that a 1:4 dilution of the serum results in a peak current (due to the nonspecific ferrocyanide leakage) of less than 0.5 nA over the background. A greater concentration of serum can result in as much as 86% of the entrapped marker leaking from within the membrane prior to a lysing event.

The linear response of ferrocyanide release as a function of IgM content in the sample is illustrated in figure 3. From these results the inefficiency of this particular complement lysis is evident. If the peak current resulting from a surfactant lysis of the membrane is defined as 100% release of the marker, the response of the IgM-saturated DNP-liposomes (between 100 and 200 *μ*L of IgM supernate) implies that only 6–10% of the marker is released. Future research using this technique will include investigating alternate complement sources (such as guinea pig) which will hopefully prove to be more reactive and improve the sensitivity of the assay.

## Figures and Tables

**Figure 1:**
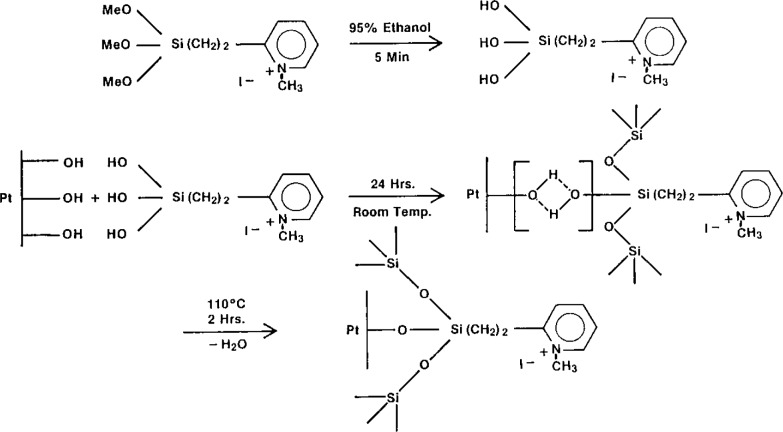
Reaction sequence for fabrication of silane-modified electrodes with approximately 100% of the sites methylated.

**Figure 2:**
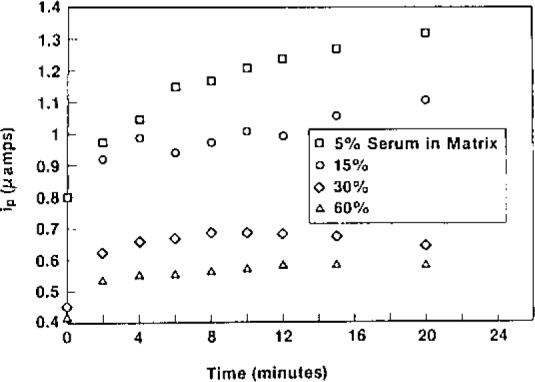
Ferrocyanide uptake for 1.2 *μ*m thick polymer modified electrodes for increasing amounts serum in the sample matrix.

**Figure 3:**
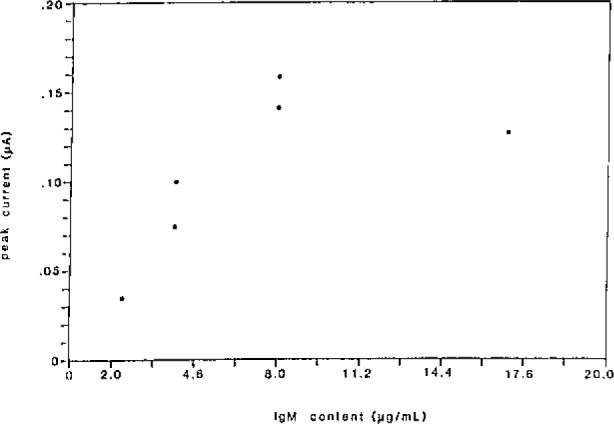
Analytical response (working) curve for DNP/Liposomes. Voltammograms were recorded at a modified electrode versus a Ag wire quasi reference electrode. Samples were incubated for 30 minutes at 37 °C and the released ferrocyanide preconcentrated for 30 minutes prior to scan.
